# What is the role of checkpoint inhibitors in neuroendocrine neoplasms?

**DOI:** 10.18632/oncotarget.27768

**Published:** 2020-10-20

**Authors:** Taymeyah Al-Toubah, Eleonora Pelle, Jonathan Strosberg

**Keywords:** neuroendocrine neoplasm, neuroendocrine tumor, neuroendocrine carcinoma, immunotherapy, PD-1 inhibitor

Neuroendocrine neoplasms (NENs) are clinically and biologically heterogeneous malignancies that can originate in multiple organs, including the gastrointestinal tract, pancreas, and lungs [1]. They are subdivided into well-differentiated neuroendocrine tumors (NETs) and poorly differentiated neuroendocrine carcinomas (NECs). NETs are often, but not always, slow growing and characterized by a relatively low tumor mutational burden (TMB), with few genetic aberrations typically involving chromatin remodeling genes such as MEN, DAXX, and ATRX [2, 3]. However, additional mutations can develop over time, potentially due to the influence of cytotoxic treatments [4, 5]. NET cells tend to express somatostatin receptors (SSTRs), a biological characteristic that has been exploited therapeutically in the development of ‘cold’ and radiolabeled somatostatin analogs. NECs, on the other hand, are invariably aggressive and tend to carry a higher burden of somatic mutations, often involving common oncogenes and tumor suppressor genes such as p53, Rb1, and RAS [6–8].

Studies of immune checkpoint inhibitors (ICIs), including inhibitors of PD-1/PD-L1 and CTLA-4, have come relatively late to the field of NENs; however, multiple trials have finally reported outcomes in recent years, allowing for the development of preliminary insights on the role of ICIs. One important finding is that a single-agent PD-1 inhibitor is relatively ineffective in treating both well-differentiated NETs and poorly differentiated NECs. Several studies support this conclusion. One is the phase II Keynote 158 (KN158) study of pembrolizumab in rare tumors, including a well-differentiated NET arm [9]. Among 107 patients with well-differentiated NETs originating in the gastrointestinal tract, pancreas, and lungs, only four patients (3%) achieved an objective partial radiographic response (PR): 3 pancreatic NETs and one with a rectal NET. The overall rate of PD-L1 expression by combined positive score (CPS) score, with PD-L1 positive defined as CPS ≥ 1, was 15.9%. However, it was 0% among the four responding patients, suggesting that this biomarker should not be considered predictive for ICI therapy in NETs. It is also noteworthy that none of the small intestinal NETs experienced a radiographic response, possibly due to their remarkably low tumor mutational burden (TMB).

Another study of a PD-1 inhibitor, spartalizumab, consisted of 4 cohorts of roughly 30 patients each: GI NETs, pancreatic NETs, lung NETs (typical and atypical lung carcinoids), and poorly differentiated NECs of any primary site except lung (e.g., small cell lung cancer) and skin (Merkel cell) [10]. This study reported low response rates (< 10%) in all cohorts, except for a 20% response rate among lung NETs, particularly within the atypical carcinoid group. However, even within this cohort, durations of response were relatively short on average, and investigation of this drug in NETs was not expanded.

As a single agent, pembrolizumab was studied in a population of high-grade NENs: poorly differentiated NECs and well-differentiated grade 3 NETs [11]. Among 29 patients studied, only 1 with an esophageal NEC responded, again confirming the relative inefficacy of single-agent PD-1 inhibition in NENs. Median PFS was 8.9 weeks, and the median OS was 20.4 weeks.

Studies of combination immunotherapy consisting of PD-1 inhibitors and CTLA-4 inhibitors have been more promising. The Dual Antibody in Rare Tumors (DART) study sponsored by the Southwest Oncology Group (SWOG) included two NEN cohorts with relatively broad and overlapping inclusion criteria [12]. An analysis of one of the cohorts revealed that objective responses occurred in 8 out of 18 (44%) patients with high-grade NETs/NECs (including several lung NECs) but in 0 out of 14 low and intermediate-grade NETs (0%). Although demonstrating encouraging efficacy in high-grade disease, it is essential to emphasize that this was an unplanned subset analysis. Indeed, recently reported results of a Spanish study evaluating the combination of durvalumab (PD-L1 inhibitor) and tremelimumab (CTLA-4 inhibitor) in 4 cohorts (gastrointestinal NET, pancreatic NET, lung NET, and grade 3 NET/poorly differentiated NEC) demonstrated much lower response rates of < 10% in all cohorts, including the high-grade NET/NEC cohort where the immune-related overall response rate (irORR) was 9.4% [13]. PD-L1 expression did not appear to be predictive of response in most cohorts except for cohort 1 (lung NETs).

One might inquire about the role of chemoimmunotherapy in NECs, particularly in light of phase III studies in small cell lung cancer demonstrating modestly improved OS with the addition of either atezolizumab or durvalumab to platinum-based chemotherapy [14, 15]. The answer is that, thus far, there are no data to support this practice in extrapulmonary NECs, including extrapulmonary small cell carcinomas of the esophagus, bladder, cervix, prostate, and other sites. It is also important to note that small cell lung cancer is uniquely associated with smoking, a factor that likely influences its mutational burden and neoantigen landscape [16]. Therefore, it is unclear whether lessons from small cell lung cancer can be applied to extrapulmonary NECs.

In summary, it appears that single-agent PD-1 inhibitors are relatively inactive in both well-differentiated NETs (especially in small bowel NETs) and poorly differentiated NECs. On the other hand, the role of combined inhibitors of PD-1 or PD-L1 and CTLA-4 is less certain. While combination immunotherapy appears to be relatively inactive in well-differentiated low and intermediate-grade NETs, it may have a higher efficacy rate in high-grade NETs and NECs. One study of combination ICI therapy showed a response rate of 44% in an unplanned analysis of a small subset, and another, prospectively defined study showed a response rate of 9%. It is likely the true response rate is closer to the latter. However, even a response rate as low as 10% may be sufficient to justify the use of combination immunotherapy as salvage treatment in NEC patients who have few treatment options beyond first-line platinum-based chemotherapy. Certain known predictive factors, such as microsatellite instability (MSI) and high TMB, can identify optimal ICI therapy candidates. In one study, MSI high frequency was 4% and mean TMB was 9.5 mut/MB in high grade NETs/NECs versus. 0% in and 5.1 mut/MB in low-intermediate grade tumors [17]. Our future challenge is to identify additional predictive biomarkers, which will help enrich the population of responding patients and spare the majority who are unlikely to respond from the toxicities of treatment.
